# Tuberculosis Disease Among Nonimmigrant Visa Holders Reported to US Quarantine Stations, January 2011–June 2016

**DOI:** 10.1007/s10903-024-01601-w

**Published:** 2024-06-05

**Authors:** Laura A. Vonnahme, Kate M. Shaw, Reena K. Gulati, Michelle R. Hollberg, Drew L. Posey, Joanna J. Regan

**Affiliations:** Division of Global Migration and Quarantine, Centers for Disease Control and Prevention, Atlanta, GA USA

**Keywords:** Travelers, Migration, Visa status, Overseas screening, Tuberculosis

## Abstract

US-bound immigrants and refugees undergo a mandatory overseas medical examination that includes tuberculosis screening; this exam is not routinely required for temporary visitors applying for non-immigrant visas (NIV) to visit, work, or study in the United States. US health departments and foreign ministries of health report tuberculosis cases in travelers to Centers for Disease Control and Prevention Quarantine Stations. We reviewed cases reported to this passive surveillance system from January 2011 to June 2016. Of 1252 cases of tuberculosis in travelers reported to CDC, 114 occurred in travelers with a long-term NIV. Of these, 83 (73%) were infectious; 18 (16%) with multidrug-resistant tuberculosis (MDR TB) and one with extensively drug-resistant tuberculosis (XDR TB). We found evidence that NIV holders are diagnosed with tuberculosis disease in the United States. Given that long-term NIV holders were over-represented in this data set, despite the small proportion (4%) of overall non-immigrant admissions they represent, expanding the US overseas migration health screening program to this population might be an efficient intervention to further reduce tuberculosis in the United States.

## Background

In 2017, 70% of reported tuberculosis (TB) cases in the United States occurred among non-US-born persons, with the case rate among non-US-born persons estimated to be 15 times higher (14.6 per 100,000) than among US-born persons (1 per 100,000) [1], although the majority (> 80%) of U.S. TB cases result from reactivated latent TB infection (LTBI). The Immigration and Nationality Act requires overseas medical exams for US-bound immigrants (defined as people applying for US Lawful Permanent Resident (LPR) status overseas), status adjusters (defined here as people applying for LPR status in the United States) and refugees [2]. CDC issues the medical exam requirements, called Technical Instructions (TIs), used by overseas panel physicians who are authorized by the Department of State to perform the immigration medical examination. In the United States, Civil Surgeons, physicians designated by the Department of Homeland Security’s U.S. Citizenship and Immigration Services, follow similar Technical Instructions to complete the medical exam for status adjusters. In 2007, CDC revised the TB TIs to include the use of Mycobacterium tuberculosis culture, drug susceptibility testing (DST), and directly observed therapy (DOT) [3]. The implementation of culture, DST, and DOT has aided in reducing TB cases among newly-arriving non–US-born individuals in the United States [4–6].

Currently only US-bound immigrants, status adjusters and refugees are required to undergo an immigration medical exam that includes screening for TB [3]. In 2015, of the 182 million foreign nationals counted as visa admissions to the United States, only 1% (approx. 1 million) were immigrants, status adjusters or refugees; the majority (99%) entered as temporary visitors, including persons with non-immigrant visas (NIV) issued overseas (Table 1) [7]. A subset of NIVs issued for students, workers, cultural exchange visitors, and trainees allow individuals to reside in the United States for 6 months or longer as a long-term visitor; on average more than 6 million long-term NIVs are issued annually [7].

**Table 1:** Estimated Admissions to the U.S. in 2015 and TB cases in travelers by immigration status and visa type

Immigration status	Visa types	Estimated Arrivals to U.S. in 2015 [10] N (% within Immigration status)	TB cases in travelers 2011–2016 N (% within Immigration status)
Total		~ 182,300,000	440
Non-immigrant	Visitors exempted from I-94/I-94W forms^a^	104,400,000 (66)	0
	Foreign government officials/diplomats (A, G & NATO)	438,477 (0.3)	5 (1.5)
Short-term visas	Visitors for business or pleasure/tourism (B1, B2)	46,605,955 (29)	141 (41)
	Transit aliens or crewmembers (C, D)	689,990 (0.4)	61 (18)
Long-term visas	Students (F & M)	1,990,661 (1)	71 (21)
	Workers, exchange visitors & trainees (H, I, J, L, O, P, Q, R, TD)	4,298,890 (3)	43 (13)
	Victims of human trafficking or crime (U, T)	Unknown	0 (0%)
	Unknown	–	18 (5.5)
	Total estimated non-immigrant arrivals	158,423,973 (87% of total)	339 (77% of total)
Immigrants and Status adjusters	Family-sponsored	213,910 (24)	5 (10)
	Immediate relative immigrants of US citizens (IR 1, 2, 5)	465,068 (52)	11 (23)
	Employment-sponsored immigrants (E)	144,047 (16)	1 (2)
	Diversity – Lottery immigrants	47,934 (5)	5 (10)
	Parolees	23 (< 0.01)	
	Other	28,054 (3)	4 (8)
	Unknown	22 (< 0.01)	22 (46)
	Total estimated immigrant arrivals	899,058 (0.5% of total)	48 (11% of total)
Travelers from visa-waiver country^b^		22,419,941 (12% of total)	13 (3% of total)
Refugee		118,431 (0.06% of total)	29 (6.5% of total)
Asylees		33,564 (0.02% of total)	11 (2.5% of total)

^a^Majority are business travelers or tourists from Canada and Mexico

^b^38 countries are a part of the visa-waiver program

NIV holders, and specifically those with longer-term visas, may be diagnosed with TB while in the United States. A study in 2012 estimated the TB rate among long-term visitors from high-incidence TB countries to be 60.9 per 100,000, compared to a rate of 3.2 per 100,000 in the U.S. general population [8, 9]. Another study estimated the TB rate to be 48.1 per 100,000 among non–US-born student visa holders [10]. A large multisite study conducted by the TB Epidemiologic Studies Consortium showed that temporary visa holders accounted for 22% of TB cases reported in the United States during 2005–2006 among newly arrived non–US-born individuals 15 years or older [9]. In the same study, temporary visa holders with pulmonary TB disease were 1.6 times as likely to have smear-positive sputa at diagnosis when compared to individuals with LPR status with pulmonary TB disease, and approximately one-fourth of cases reported being symptomatic at or before admission to the United States. Previous literature suggests that instituting requirements for overseas screening and treatment for specific long-term NIV holders may reduce US TB diagnoses and result in an estimated cost saving of $2.7 million annually [8–11].

The US national TB surveillance system does not require the collection of information on visa status. The objective of our analysis was to describe the cohort of confirmed cases of TB disease among travelers reported in the CDC Quarantine Activity Reporting System (QARS), a passive surveillance system maintained by the 20 CDC Quarantine Stations at US ports of entry [12]. The purpose of this database is to collect information about persons who might have been infectious during travel or intended to travel while infectious; it was not designed to collect information on all TB cases in the United States. However, it is the only US federal data source that includes visa status of persons with TB disease. We examined TB cases in QARS to better understand the extent to which NIV holders are diagnosed with TB in the United States.

## Methods

State, local, and territorial health departments and foreign ministries of health notify CDC Quarantine Stations of possible or confirmed cases of TB in travelers or in patients likely to travel. The CDC has criteria for reporting cases among persons who have recently traveled internationally, including traveling while having infectious drug susceptible, multi-drug resistant (MDR), or extensively drug-resistant (XDR) TB as defined by Marienau et al. [13]. MDR TB is defined as resistance to at least isoniazid and rifampin and XDR TB is resistance to isoniazid and rifampin plus a fluoroquinolone and at least one of three injectable second-line drugs (i.e., amikacin, kanamycin, or capreomycin). Travel restrictions apply to persons likely to travel while infectious [17]. CDC stores the reported travel health data, including demographic information, country of birth, admission status and visa type and clinical and laboratory case data in QARS, a secure, restricted-access database [12], and uses the data to determine whether public health actions are needed to prevent spread of travel-related disease [14, 15]. Although these were the recommended reporting criteria, all TB disease cases recorded in QARS were included in this analysis.

We reviewed de-identified data for all confirmed cases of TB disease reported to CDC Quarantine Stations and recorded in QARS from January 1, 2011, to June 30, 2016. A confirmed case of TB disease was defined as laboratory-confirmed by culture or polymerase chain reaction (PCR) or a clinical diagnosis [16]. Variables extracted included demographic information, country of birth, admission status and visa type, and diagnostic and laboratory results. TB infectiousness at the time of diagnosis was defined here as having a positive AFB smear or a cavitary lesion on chest x-ray, or both. Drug susceptibility test (DST) results were categorized as susceptible to all first-line drugs, mono-resistant to one first-line drug, MDR or XDR. We completed a manual review of all case reports to improve data quality of immigration status and visa variables.

Cohort data were stratified by reported admission status which represented an individual’s visa type or refugee status at time of report. QARS has 12 admission status categories: US citizen, lawful permanent resident (LPR), NIV holder, immigrant visa holder, refugee, asylee, visa-waiver traveler, unaccompanied minor, individual in transit or not entering the United States, undocumented or illegal, unknown, or other. US citizens born in the United States or with an unknown country of birth, and individuals in transit or not entering the United States were excluded from further analysis.

Among the sub-cohort of NIV holders, we reported demographics, country of birth, infectiousness, and DST results by long-term and short-term NIV holders. Visas for students (F and M), specialty or temporary workers (H), exchange visitors & trainees (I, J, L, O, P, Q, R and TD), and victims of human trafficking or crime (U and T) were categorized as long-term NIV holders. Visitors for business or pleasure/tourism (B1 and B2) and transit aliens or crewmembers (C and D) were categorized as short-term NIV holders. Visas for foreign government official or diplomats (A, G and NATO), were categorized as other NIV holders; individuals without a specified NIV (unknown) in QARS were also included in the other category [17].

Analysis was performed in SAS version 9.3. This project was reviewed in accordance with CDC institutional review policies and procedures and was determined to be non-research.

## Results

**Fig. 1:**
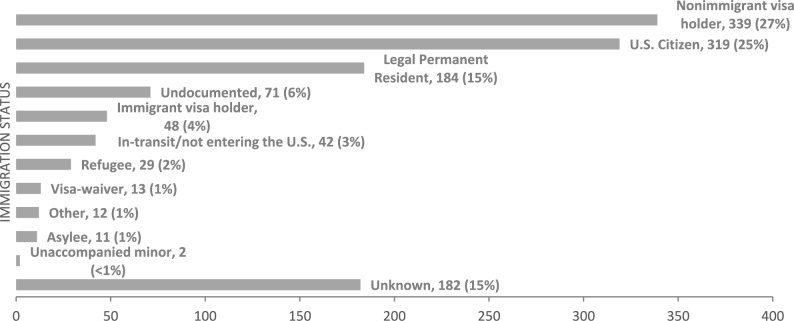
Immigration status of travelers with TB disease reported to CDC Quarantine Stations, 1 Jan 2011–1 Jul 2016

1252 cases of confirmed TB disease in travelers were reported to CDC Quarantine Stations during the cohort time frame. Of these cases, the majority (n = 858, 68.5%) were reported after travel occurred, while the rest were reported before travel 315 (25.2%), during travel 77 (6.2%) or unknown 2 (0.2%). The form of travel being used were flights in most cases (n = 1021, 81.5%), followed by land transportation (n = 142, 11.3%), ships (n = 75, 6.0%) or unknown (n = 14, 1.1%). Nonimmigrant visas represented the largest proportion (n = 339, 27%) of cases overall, followed by US citizens (n = 319, 25%) and Lawful Permanent Residents (n = 184, 15%) (Fig. 1). Among the US citizens, 26% (n = 83) were born outside the United States, 8% (n = 26) were born in the United States, and 66% (n = 210) had an unknown country of birth; those born in the United States or with an unknown country of birth (n = 236) were excluded from further analyses. Travelers in-transit or not entering the United States (n = 42, 3%) were also excluded from further analysis. We classified 974 (78%) travelers as non–US-born based on reported immigration status and/or visa or reported country of birth if the individual reported US citizenship. Among this sub-cohort of individuals with TB disease who were classified as non–US-born, the majority (78%, n = 759) were smear positive or had cavitary lesion(s) on chest x-ray indicating a high degree of infectiousness.

Among non-immigrant visa holders with TB disease, the largest proportion (41%, n = 141) were traveling with a short-term visa for business or pleasure/tourism, followed by (21%, n = 71) with a long-term visa for students (Table 1). Overall, long-term NIV holders comprised (34%, n = 114) of TB disease cases among non-immigrant admissions in this dataset (Table 2). India, Philippines, China, Indonesia and Nigeria were the top five countries of birth reported by non-immigrants with TB disease; all five countries are listed on the WHO list of high-burden countries for TB [18]. 68 unique countries of birth were reported. The median age for short-term NIV holders with TB disease was 42 years old and 62% were male. Long-term NIV holders had a median age of 24 years old and 47% were male (Table 2).

**Table 2:** Demographic characteristics, TB infectiousness and drug susceptibility among non-immigrant admissions^a^ with TB disease reported to CDC Quarantine Stations, 1 Jan 2011–1 Jul 2016

	N (%)	Long-term visas^a^ N (%)	Short-term/visitor visas^b^ N (%)	Other^c^ N (%)
Total	339	114 (34)	202 (59)	23 (7)
Gender^d^				
Female	147 (43)	60 (53)	75 (37)	12 (52)
Male	191 (56)	54 (47)	126 (62)	11 (48)
Age at time of report (yrs)				
Median	32	24	42	35
< 5	0	–	–	–
5–14	1 (0.3)	1 (1)	–	–
15–24	79 (23)	60 (53)	17 (8)	2 (9)
25–44	154 (45)	49 (43)	95 (47)	10 (43)
45–64	65 (19)	3 (3)	54 (27)	8 (35)
65 +	40 (12)	1 (1)	36 (18)	3 (13)
Country of birth^e^				
India	63 (19)	29 (25)	32 (16)	2 (9)
Philippines	35 (10)	5 (4)	30 (15)	–
China	30 (9)	20 (18)	8 (4)	2 (9)
Indonesia	21 (6)	3 (3)	18 (9)	–
Nigeria	16 (5)	6 (5)	10 (5)	–
Vietnam	15 (4)	5 (4)	10 (5)	–
Mexico	11 (3)	–	11 (5)	–
Pakistan	10 (3)	4 (3)	6 (3)	–
South Africa	9 (3)	4 (3)	5 (2)	–
Other^f^	129 (38)	38 (33)	72 (36)	19 (82)
Infectiousness				
Smear ( +) and/or cavitary Chest X-Ray (CXR)	263 (78)	83 (73)	162 (80)	18 (78)
Smear (-) and non-cavitary^g^	45 (13)	21 (18)	21 (10)	3 (13)
Smears (-) or unknown AND non-cavitary or unknown CXR^g^	31 (9)	10 (9)	19 (10)	2 (8)
Drug Susceptibility				
Susceptible to all 1st-line drugs	199 (59)	72 (63)	118 (58)	9 (39)
Monoresistant to one 1st-line drug	36 (11)	12 (11)	22 (11)	2 (9)
MDR^h^	37 (11)	18 (16)	18 (9)	1 (4)
XDR^i^	4 (1)	1 (1)	2 (1)	1 (4)
Unknown/Unavailable	63 (19)	11 (10)	42 (21)	10 (43)

^**a**^F & M—^a^Students; H, I, J, L, O, P, Q, R, TD—Workers, exchange visitors & trainees; U, T—Victims of human trafficking or crime

^b^B1, B2—Visitors for business or pleasure/tourism; C, D—Transit aliens or crewmembers

^c^A, G & NATO – Foreign government officials/diplomats; unknown

^d^One individual with unknown gender

^e^If country of birth was unknown, it was assumed that country of birth was the passport country, except if passport country was the U.S

^f^59 unique countries

^g^Among these 76 travelers, 89% were culture positive; due to reporting criteria these categories are likely underrepresented

^h^Multidrug-resistant to isoniazid and rifampin

^i^Extensively drug-resistant TB

Most non-immigrant TB disease cases had culture-confirmation (87%, n = 296); 9% (n = 29) had unknown culture results and 4% (n = 14) were culture negative and assumed to have clinical diagnosis of TB. The majority of the non-immigrant cases in this cohort (78%, n = 263) were smear positive or had cavitary lesion(s) on a chest x-ray (CXR), indicating infectiousness. While 73 (21%) were smear negative and/or had no cavitary lesions on a chest x-ray, the majority (n = 65, 89%) were still culture positive for M. tuberculosis and could have also been infectious. The majority of non-immigrant cases in the cohort were susceptible to all first-line drugs (59%). However, 37 (11%) were confirmed MDR-TB cases and 4 (1%) were confirmed XDR cases; 33 (10%) individuals in the cohort were mono-resistant to either isoniazid or rifampin, and 3 (1%) were susceptible to isoniazid and rifampin but resistant to one or more other first line drugs.

## Discussion

To determine how to best implement medical screening for TB overseas, it is important to know who is at risk. Given the enormous number of travelers to the United States each year (182,300,000 reported in 2015, Table 1), it is impossible to screen all travelers for TB prior to arrival in the United States. Resources must be focused on those most at risk for being diagnosed with TB disease in the United States. Overseas screening aids in the elimination of TB not only by diagnosing and treating people for TB disease before departing for the United States but also identifying people at risk for TB disease and referring them for further follow-up in the United States [6]. Although the immigrant visa screening process has aided in decreasing TB diagnoses in non-US-born individuals, recent studies suggest that expanding screening and treatment for international travelers at higher risk for TB could aid in further decreasing US incidence [8–11].

Passive surveillance also detected TB cases among LPR and non-US-born US citizens (n = 267). Although these travelers might have received a TB screening at some point, it could have been years and possibly decades in the past and could have occurred overseas or domestically. Non-U.S.-born persons may also be exposed to someone with TB disease during travel after having emigrated to the U.S. Liu et al. [8] demonstrated a low rate of TB disease among immigrants and refugees who had recently received overseas screening; thus, these cases might be due to missed domestic follow-up, progression of latent TB infection, or infection after initial TB screening.

The QARS database represents an important subset of TB cases. These are cases of TB in persons who traveled or are planning to travel and who were reported to CDC to prevent transmission of TB disease. In this cohort, long-term visitors made up 34% of the NIV holder TB disease cases reported to QARS and 4% (6,289,551) of total annual non-immigrant arrivals to the United States. Arrivals with short-term NIV visas accounted for 59% of NIV TB cases in this cohort, however they accounted for a much larger number of yearly arrivals (46,605,955). Although the data have limitations, long-term NIV holders appear to comprise a small percentage of overall admissions, while contributing significantly to domestically diagnosed TB disease, including MDR and XDR cases. These data suggest that screening long-term NIVs for TB disease before they depart for the United States could increase diagnoses of TB disease among travelers to the U.S.

As expected, most of these cases occurred in travelers from countries with a TB rate of greater than 20 cases per 100,000 population. Currently, overseas TB screening is only required for immigrants and refugees. It follows that if screening were expanded to long-term NIV applicants, larger numbers of people could be identified and treated for TB disease. In 2015, the US Government published the National Action Plan for Combatting Antibiotic-Resistant Bacteria [9, 11, 19, 20]. This plan includes a provision for expanding the overseas screening program for persons migrating from countries with a high incidence of TB and MDR-TB. The results of this analysis suggest that NIV holders are being diagnosed with drug susceptible and drug-resistant TB in the United States. Although from a small, passive reporting system, these results support developing strategies to expand the US screening program to increase testing and treatment of TB disease, preventing further transmission. Although transmission of TB during air travel is not common [13, 21], it is possible, and can contribute to international spread of the disease [22–25]. Diagnosing and treating additional cases overseas before persons travel might also prevent risk of transmission of TB during international travel.

The cohort represents a limited dataset based on CDC criteria for conducting aircraft or ship contact investigations and the use of federal public health travel restrictions for travelers with suspected or confirmed TB and, therefore, may not be generalizable to all NIV holders. Typically, infectious cases, as determined by laboratory testing and treatment history, are more likely to be reported to CDC Quarantine Stations, as aircraft contact investigations are limited to cases with positive sputum smears and cavitary disease, or MDR status. Although cases that are M. tuberculosis culture positive, smear negative, and lacking cavitary lesions on chest x-ray typically make up a significant proportion of the TB disease cases in most populations, they are likely underrepresented in this database due to reporting criteria based on disease transmission during travel [15]. In addition, aircraft contact investigations are limited to flights of 8 h or greater and are only conducted if CDC is notified within 3 months of travel. Therefore, travelers from countries with flights that are under 8 h (e.g., Caribbean, South and Central America) or travelers who are diagnosed after longer periods in the United States are likely underrepresented. Use of federal public health travel restrictions for TB requires evidence of infectiousness plus noncompliance or intent to travel against public health recommendations [26]. These reporting criteria likely led to an overestimate of the proportion of cases in travelers that are infectious. Most TB patients do not travel or plan travel while infectious and would not be reported to DGMQ or captured in this database. The prevalence of TB disease among NIVs is likely higher than reported here, due to these limitations in the way the data were collected. However, we can’t determine from this dataset the amount of time from their first trip to the US (when they would receive overseas screening if that program were initiated) and when they were first diagnosed with TB in the US. So some of these cases might not have had signs or symptoms of active TB disease at the proposed screening time, though many would likely have had a positive chest x-ray, or positive IGRA if not yet culture positive, and could have been identified for further follow up in the US.

The overseas screening will occur at the travelers expense, and if a traveler needs sputum testing for smears and cultures or treatment, their processing will be delayed. This might prove challenging for travelers attempting to start work or school in the US, however, a delayed diagnosis of TB disease can also be disruptive to school and work after arrival, effecting the health of the traveler and potentially others. This process will also uncover a larger population of travelers with abnormal findings and positive IGRA results leading to an increase in the number of people identified for follow-up at US Health Departments. Although US Health Departments contact and treat thousands of people for whom they receive notifications from current overseas screening, additional strategies are needed to meet the current and proposed increased demand for these services domestically [27].

## New Contribution to the Literature

This cohort from the QARS database represents a unique, mobile population of people with TB. Unlike many data sources, QARS includes visa status at time of TB diagnosis. These findings suggest that overseas screening of specific long-term visitors, especially students and workers from countries with a high incidence of TB, would protect travelers and reduce US TB incidence.
